# Crystal structure of a new mol­ecular salt: 4-amino­benzenaminium 5-carb­oxy­penta­noate

**DOI:** 10.1107/S2056989018000737

**Published:** 2018-01-26

**Authors:** Risha Mishra, Krishnan Rangan, Raghavaiah Pallepogu

**Affiliations:** a Department of Chemistry, Dr. Harisingh Gour University, Sagar, MP 470 003, India; b Birla Institute of Technology & Science, Pilani Hyderabad Campus, Jawahar Nagar, Shamirpeet Mandal, Ranga Reddy District, Secunderabad, Andhra Pradesh 500 078, India

**Keywords:** crystal structure, *p*-phenyl­enedi­amine, adipic acid, mechanochemical synthesis, LAG, partial protonation, hydrogen bonding

## Abstract

The asymmetric unit of the title mol­ecular salt (systematic name: 4-amino­anilinium 5-carb­oxy­penta­noate), C_6_H_9_N_2_^+^·C_6_H_9_O_4_^−^, consists of half a 4-amino­benzenaminium cation (4-ABA) and half a 5-carb­oxy­penta­noate anion (5-CP); the other half of each ion is generated by inversion symmetry. Protonation of *p*-phenyl­enedi­amine (PPDA) leads to the formation of a 1:1 salt, but scrutiny of the crystal structure reveals that both of the amine groups of PPDA are partially protonated, with a half-occupied H atom. For the 5-CP anion, an H atom is positioned on an inversion center midway between two O atoms of inversion-related 5-CP ions. In the crystal, the 5-CP anions are linked by the O—H⋯O hydrogen bond to form chains propagating along the [1-10] direction. The chains are linked *via* N—H⋯O and N—H⋯N hydrogen bonds involving the 4-ABA cations, forming a three-dimensional supra­molecular framework. The title salt was also prepared by mechanochemical synthesis using liquid-assisted grinding (LAG). Its PXRD pattern matches that of the simulated pattern of the crystal structure of the title mol­ecular salt.

## Abstract 2

The asymmetric unit of the title mol­ecular salt, consists of half a 4-amino­benzenaminium cation and a half a 5-carb­oxy­penta­noate anion. Each ion lies about an inversion centre, the other half being generated by inversion symmetry. In the crystal, charge-assisted O—H⋯O, N—H⋯O and N—H⋯N hydrogen bonds together lead to the formation of a three-dimensional supra­molecular framework.

## Chemical context   

*p*-Phenyl­enedi­amine (PPDA) has been widely used to synthesize hair dyes, engineering polymers and composites. The coordination chemistry of PPDA is well documented (Adams *et al.*, 2011 ▸; Bourne & Mangombo, 2004 ▸). Adipic acid (AA) is an industrial chemical used to manufacture nylon and is also used in many drugs and food additives (Rowe *et al.*, 2009 ▸). A number of salts and co-crystals involving *p*-phenyl­enedi­amine have been reported (Thakuria *et al.*, 2007 ▸; Delori *et al.*, 2016 ▸), and adipic acid is also widely known as a co-former in co-crystal formation (Swinton Darious *et al.*, 2016 ▸; Lemmerer *et al.*, 2012 ▸; Lin *et al.*, 2012 ▸; Matulková *et al.*, 2014 ▸; Thanigaimani *et al.*, 2012 ▸). A 2:1 salt of 4-amino­anilinium (PPDAH) and sebacate, and a 1:1 salt of PPDAH and di­hydrogen trimesate have been reported recently (Delori *et al.*, 2016 ▸). We have previously reported various salts of *o*-phenyl­enedi­amine with aromatic carb­oxy­lic acids (Mishra & Pallepogu, 2018 ▸). Herein, we report on the synthesis and crystal structure of the 1:1 salt formed between *p*-phenyl­enedi­amine and adipic acid, (I).
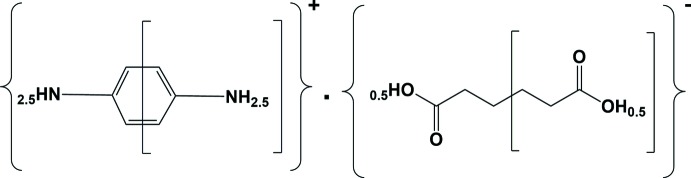


## Structural commentary   

**Figure 1:**
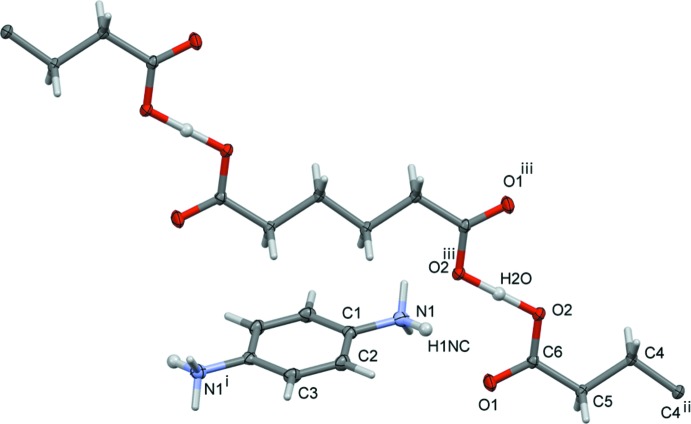
The mol­ecular structure of the title mol­ecular salt (I), with atom labelling for the asymmetric unit. Displacement ellipsoids are drawn at the 50% probability level. [Symmetry codes: (i) −*x* + 1, −*y* + 2, −*z* + 2; (ii) −*x* + 2, −*y*, −*z* + 1; (iii) −*x* + 1, −*y* + 1, −*z* + 1].

The asymmetric unit of the title salt (I), illustrated in Fig. 1 ▸, consists of half each of a 4-amino­benzenaminium cation (4-ABA) and a 5-carb­oxy­penta­noate anion (5-CP); both ions (space group *P* 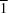) lie about inversion centres. Partial protonation (50%) has occurred at atom N1 of the cation, resulting in the formation of a salt with the formula unit C_6_H_9_O_4_^−^·C_6_H_9_N_2_^+^. One of the two adipic acid H atoms binds to atom N1 with a site-occupancy factor (SOF) of 0.5 (for atom H1*NC*), thereby positioned at two sites (because of inversion symmetry) in the cation. The other acid H atom (H2*O*) is located on an inversion center and is therefore shared equally by two O2 atoms of inversion-related anions. The C1—N1 bond length [1.4361 (13) Å] in the 4-ABA cation is longer than literature values for a non-protonated amine (C–NH_2_) group [*cf*. 1.418 (2) Å; Czapik *et al.*, 2010 ▸] and this can be attributed to the partial protonation with SOF = 0.50 at each site. In the 5-CP anion, the C6=O1 and C6—O2 bond lengths [1.2379 (12) and 1.2802 (11) Å, respectively] are similar to the values reported for 2-methyl­imidazolium hydrogen adipate monohydrate [1.244 (2) and 1.264 (2) Å, respectively; Meng *et al.*, 2009 ▸] in which a carb­oxy­lic acid H atom is also statistically distributed between the two carb­oxy groups and a hydrogen-bonded chain is formed. In (I), the position of this H atom (H2*O*) was located in a difference-Fourier map and found to be situated on an inversion centre (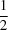, 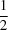, 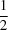). It is positioned symmetrically between two O2 atoms of two inversion-related 5-CP ions, which accounts for the long O—H bond length of 1.22 Å (see Table 1 ▸). The C4^ii^—C4—C5—C6 torsion angle of −179.82 (9)° indicates that the carbon chain in the anion is fully extended [see Fig. 1 ▸ for symmetry code (ii)].

## Supra­molecular features   

**Figure 2:**
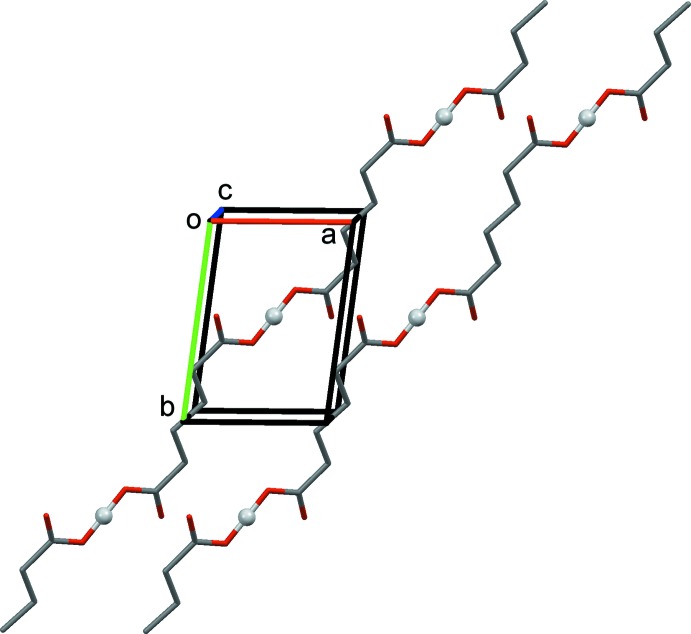
A view along the *c* axis of the O—H⋯O hydrogen-bonded chain of 5-CP anions (see Table 1 ▸). The H atoms (H2*O*; shown as grey balls) are shared between O2 atoms of inversion-related anions. The C-bound H atoms and the cations have been omitted.

**Figure 3:**
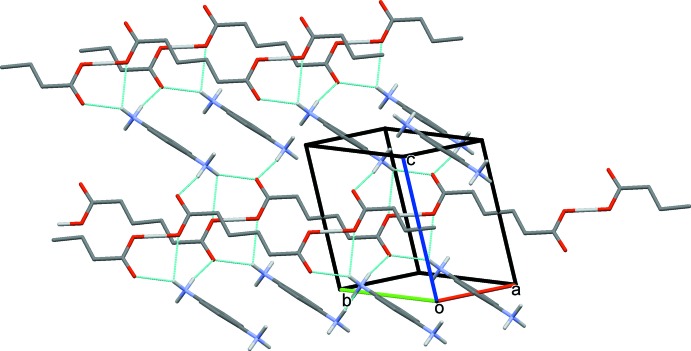
A partial view, normal to the *ab* plane, of the crystal packing of the title mol­ecular salt (I). Hydrogen bonds are shown as dashed lines (see Table 1 ▸), and C-bound H atoms have been omitted for clarity.

**Figure 4:**
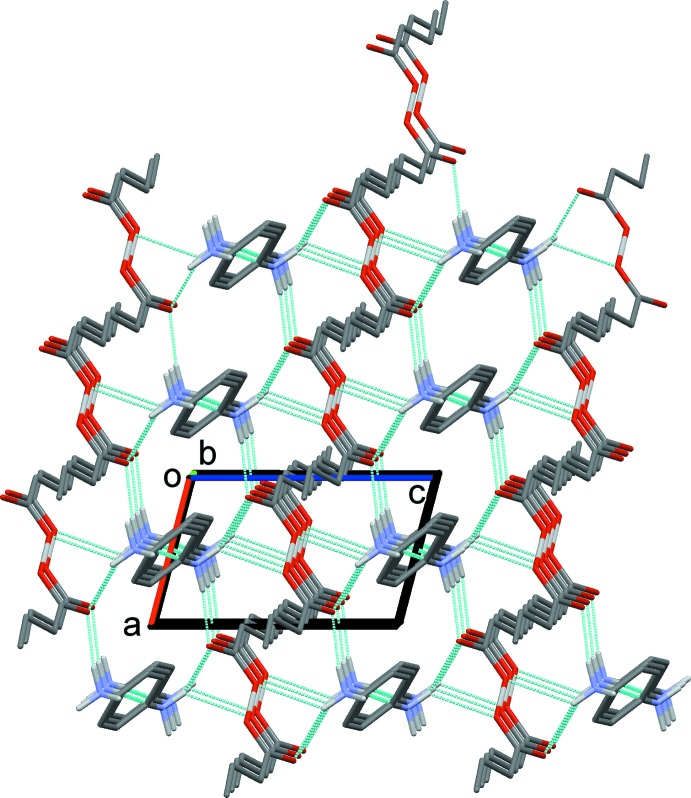
A view along the *b* axis of the crystal packing of the title mol­ecular salt (I), showing the three-dimensional supra­molecular framework. Hydrogen bonds are shown as dashed lines (see Table 1 ▸), and C-bound H atoms have been omitted for clarity.

In general, adipic acid (AA) forms 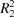
(8) homosynthons facilitated by strong O—H⋯O inter­actions; however, it is known to form heterosynthons when co-crystallized with amines (Lemmerer *et al.*, 2010 ▸, 2012 ▸). In the crystal structure of (I), the formation of the 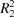
(8) homosynthon is not favoured because of the inversion-related arrangement of the 5-CP anions, which forms a linear chain along the [1
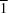
0] direction (Fig. 2 ▸, Table 1 ▸) as a result of the sharing of the H atom (H2*O*) between the O2 atoms of inversion-related anions as discussed above; otherwise this could be supposed to be the contribution of a strong O—H⋯O hydrogen bond. The crystal structure features inter­ionic N—H⋯O and O—H⋯O hydrogen bonds between the anions and cations (Table 1 ▸, Fig. 3 ▸). One of the H atoms (H1*BA*) on N1 is a bifurcated hydrogen-bond donor and the carboxyl­ate O1 atom of the anion acts as a triple acceptor, accepting H atoms from two different NH_3_ groups (H1*NA* and H1*NB*), as illustrated in Fig. 3 ▸. The chains of the 5-CP anions are linked *via* the N—H⋯O, O—H⋯O and N—H⋯N hydrogen bonds, forming a three-dimensional supra­molecular framework (Table 1 ▸ and Fig. 4 ▸).

## Database survey   

A search of the Cambridge Structural Database (CSD, Version 5.38, update May 2017; Groom *et al.*, 2016 ▸) for 4-amino­anilinium and 1,4-phenyl­enedi­ammonium salts gave 70 hits. The crystal structure of *p*-phenyl­enedi­amine (PPDA) itself was first reported by Povetéva & Zvonkova (1975 ▸). Perhaps the most relevant hit is for the structure of bis­(4-amino­anilinium) deca­nedioate (CSD refcode IPAPAS; Delori *et al.*, 2016 ▸), one of the few salts formed with an aliphatic diacid. There are a number of structures reported of PPDA with mineral acids (Chandrasekaran, 1969 ▸; Marsh, 2009 ▸; Anderson *et al.*, 2006 ▸), and co-crystals and salts of PPDA with organic acids (Thakuria *et al.*, 2007 ▸; Delori *et al.*, 2016 ▸). Some 1:1 co-crystals of PPDA and various diols (*viz*. 1,8-octane diol, 1,10-decane diol and 1,12-dodecane diol) have also been reported (Loehlin & Okasako, 2007 ▸).

A search of the CSD for salts of adipic acid (AA) with different amines yielded 67 hits. One such structure of partic­ular inter­est, *viz*. 2-methyl­imidazolium hydrogen adipate monohydrate, has been reported twice, once at room temperature (BOTTOU: Meng *et al.*, 2009 ▸), where the same type of partial disorder is observed with the carb­oxy­lic acid H atom statistically distributed between the two carb­oxy groups and a hydrogen-bonded chain is formed. However, the low-temperature analysis at 120 K using synchrotron radiation (BOTTOU01: Callear *et al.*, 2010 ▸), describes the structure as bis­(2-methyl­imidazolium) adipate adipic acid dihydrate. In the crystal, the adipate and adipic acid mol­ecules also form a hydrogen-bonded chain. A second structure, tetra­kis­(cyto­sin­ium) di­hydrogen bis­(adipate), also exhibits the same type of disorder of the carb­oxy­lic acid H atom (OYEREQ; Das & Baruah, 2011 ▸), and in the crystal it forms a hydrogen-bonded chain.

## Synthesis and crystallization   

The title mol­ecular salt (I), was synthesized by mixing a 5 ml methano­lic solution of adipic acid (AA: 0.5 mmol, 73 mg) and 3 ml of an aceto­nitrile solution of *p*-phenyl­enedi­amine (PPDA: 0.5 mmol, 54 mg). The reaction mixture was heated to 323 K with magnetic stirring for *ca* 30 min, and then filtered and allowed to evaporate slowly at room temperature. Purple block-like crystals of (I) were obtained after 5 d (m.p. 438 K). FTIR (KBr pellet, cm^-1)^: ν 3337, 3180, 2946, 2383, 1706, 1515, 1255, 821, 743, 501, 475.

**Figure 5:**
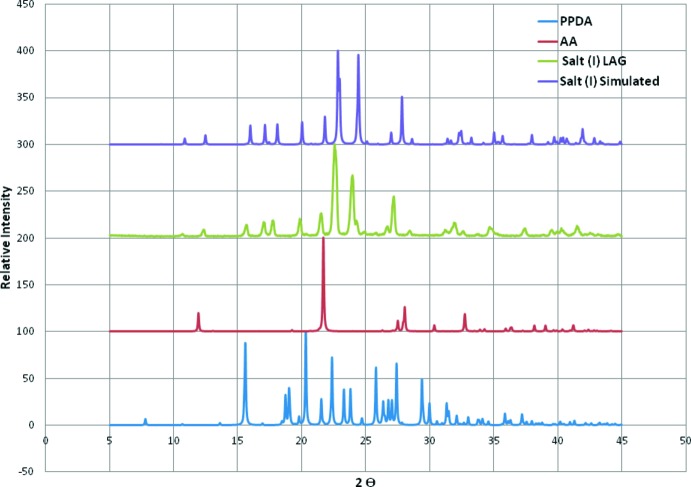
The PXRD pattern obtained from the product of the LAG experiment, and the simulated PXRD pattern of the crystal structure of the title mol­ecular salt. The PXRD patterns of the reactants used for the co-crystallization and LAG syntheses are also shown.

The title compound was also synthesized by liquid-assisted grinding (LAG). For this mechanochemical synthesis, equimolar amounts of AA (1 mmol, 146 mg) and PPDA (1 mmol, 108 mg) were ground for 20 min. in a mortar and pestle using 3 to 4 drops of aceto­nitrile. The powdered sample was collected for PXRD and the resultant pattern was scrutinized for new peaks, as evidence for the formation of the title mol­ecular salt (I), by comparing this pattern with the simulated pattern obtained from the CIF file of salt (I). The PXRD pattern of the compound obtained from the LAG experiment matches the simulated pattern obtained for (I), formed by co-crystallization (Fig. 5 ▸).

## Refinement   

Crystal data, data collection and structure refinement details are summarized in Table 2 ▸. C-bound H atoms were placed in calculated positions and refined using a riding-model approximation: 0.95–09.99 Å with *U*_iso_(H) = 1.2*U*_eq_(C). The ammonium and carboxyl H atoms were located in difference-Fourier maps and were freely refined. The H atom (H1*NC*) bound to N1 of the 4-ABA cation, with an occupancy factor of 0.5, is positioned at two sites of the cation due to inversion symmetry, giving rise to a monoprotonated species. The carb­oxy­lic acid H atom (H2*O*) is positioned symmetrically between the two O2 atoms of inversion-related 5-CP ions (H—O = 1.22 Å). This H atom (H2*O*) is located on an inversion center (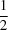, 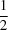, 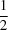) with an occupancy factor of 0.5, and hence gives rise to a mono-deprotonated species.

## Supplementary Material

Crystal structure: contains datablock(s) Global, I. DOI: 10.1107/S2056989018000737/dx2003sup1.cif

Supporting information file. DOI: 10.1107/S2056989018000737/dx2003Isup4.cml

Supporting information file. DOI: 10.1107/S2056989018000737/dx2003Isup6.mol

CCDC reference: 1816419

Additional supporting information:  crystallographic information; 3D view; checkCIF report

## Figures and Tables

**Table 1:** Hydrogen-bond geometry (Å, °)

*D*—H⋯*A*	*D*—H	H⋯*A*	*D*⋯*A*	*D*—H⋯*A*
O2—H2*O*⋯O2^i^	1.22	1.22	2.439 (1)	180
O2—H2*O*⋯O1^i^	1.22	2.45	3.178 (1)	116
N1—H1*NA*⋯O1^ii^	0.91 (2)	1.97 (2)	2.871 (1)	171 (1)
N1—H1*NB*⋯O1	0.90 (2)	2.24 (2)	3.060 (1)	152 (1)
N1—H1*NB*⋯O2^i^	0.90 (2)	2.51 (2)	3.098 (1)	123 (1)
N1—H1*NC*⋯N1^iii^	0.95 (2)	1.89 (2)	2.840 (1)	174 (2)

Symmetry codes: (i) 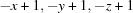; (ii) 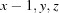; (iii) 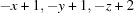.

**Table 2:** Experimental details

Crystal data	
Chemical formula	C_6_H_9_N_2_^+^·C_6_H_9_O_4_^−^
*M*_r_	254.28
Crystal system, space group	Triclinic, *P* 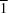
Temperature (K)	100
*a*, *b*, *c* (Å)	5.2918 (3), 7.1666 (4), 8.4205 (7)
α, β, γ (°)	92.069 (6), 104.165 (6), 97.172 (5)
*V* (Å^3^)	306.47 (4)
*Z*	1
Radiation type	Mo *K*α
μ (mm^−1^)	0.10
Crystal size (mm)	0.42 × 0.38 × 0.32

Data collection	
Diffractometer	Rigaku Oxford Diffraction XtaLAB Pro: Kappa dual offset/far
Absorption correction	Multi-scan (*CrysAlis PRO*; Rigaku OD, 2015 ▸)
*T*_min_, *T*_max_	0.924, 1.000
No. of measured, independent and observed [*I* > 2σ(*I*)] reflections	3776, 1416, 1341
*R*_int_	0.015
(sin θ/λ)_max_ (Å^−1^)	0.685

Refinement	
*R*[*F* ^2^ > 2σ(*F* ^2^)], *wR*(*F* ^2^), *S*	0.034, 0.092, 1.08
No. of reflections	1416
No. of parameters	96
H-atom treatment	H atoms treated by a mixture of independent and constrained refinement
Δρ_max_, Δρ_min_ (e Å^−3^)	0.39, −0.22

Computer programs: *CrysAlis PRO* (Rigaku OD, 2015 ▸), *SHELXS97* (Sheldrick, 2008 ▸), *SHELXL2016/6* (Sheldrick, 2015 ▸), *OLEX2* (Dolomanov *et al.*, 2009 ▸), *Mercury* (Macrae *et al.*, 2008 ▸), *PLATON* (Spek, 2009 ▸) and *publCIF* (Westrip (2010 ▸).

## supplementary crystallographic information

### Crystal data

**Table:**

C_6_H_9_N_2_^+^·C_6_H_9_O_4_^−^	*Z* = 1
*M**_r_* = 254.28	*F*(000) = 136
Triclinic, *P*1	*D*_x_ = 1.378 Mg m^−^^3^
*a* = 5.2918 (3) Å	Mo *K*α radiation, λ = 0.71073 Å
*b* = 7.1666 (4) Å	Cell parameters from 5347 reflections
*c* = 8.4205 (7) Å	θ = 5.2–29.2°
α = 92.069 (6)°	µ = 0.10 mm^−^^1^
β = 104.165 (6)°	*T* = 100 K
γ = 97.172 (5)°	Block, purple
*V* = 306.47 (4) Å^3^	0.42 × 0.38 × 0.32 mm

### Data collection

**Table:**

Rigaku Oxford Diffraction XtaLAB Pro: Kappa dual offset/far diffractometer	1416 independent reflections
Radiation source: fine-focus sealed X-ray tube, Enhance (Mo) X-ray Source	1341 reflections with *I* > 2σ(*I*)
Graphite monochromator	*R*_int_ = 0.015
ω scans	θ_max_ = 29.1°, θ_min_ = 5.0°
Absorption correction: multi-scan (CrysAlis PRO; Rigaku OD, 2015)	*h* = −5→7
*T*_min_ = 0.924, *T*_max_ = 1.000	*k* = −9→8
3776 measured reflections	*l* = −11→10

### Refinement

**Table:**

Refinement on *F*^2^	Secondary atom site location: difference Fourier map
Least-squares matrix: full	Hydrogen site location: mixed
*R*[*F*^2^ > 2σ(*F*^2^)] = 0.034	H atoms treated by a mixture of independent and constrained refinement
*wR*(*F*^2^) = 0.092	*w* = 1/[σ^2^(*F*_o_^2^) + (0.0483*P*)^2^ + 0.0998*P*] where *P* = (*F*_o_^2^ + 2*F*_c_^2^)/3
*S* = 1.08	(Δ/σ)_max_ < 0.001
1416 reflections	Δρ_max_ = 0.39 e Å^−^^3^
96 parameters	Δρ_min_ = −0.22 e Å^−^^3^
0 restraints	Extinction correction: (SHELXL-2016/6; Sheldrick, 2015), Fc^*^=kFc[1+0.001xFc^2^λ^3^/sin(2θ)]^-1/4^
Primary atom site location: structure-invariant direct methods	Extinction coefficient: 0.15 (2)

### Special details

**Table:**

Geometry. All esds (except the esd in the dihedral angle between two l.s. planes) are estimated using the full covariance matrix. The cell esds are taken into account individually in the estimation of esds in distances, angles and torsion angles; correlations between esds in cell parameters are only used when they are defined by crystal symmetry. An approximate (isotropic) treatment of cell esds is used for estimating esds involving l.s. planes.

### Fractional atomic coordinates and isotropic or equivalent isotropic displacement parameters (Å^2^)

**Table:**

	*x*	*y*	*z*	*U*_iso_*/*U*_eq_	Occ. (<1)
O1	0.88613 (16)	0.51078 (11)	0.73311 (10)	0.0284 (2)	
O2	0.62365 (13)	0.36690 (9)	0.50333 (8)	0.0159 (2)	
H2O	0.500000	0.500000	0.500000	0.079 (10)*	
C4	0.90322 (17)	0.07097 (12)	0.49487 (11)	0.0140 (2)	
H4A	0.867169	0.119848	0.384244	0.017*	
H4B	0.735161	0.007357	0.511087	0.017*	
C5	1.01023 (17)	0.23441 (12)	0.62353 (11)	0.0127 (2)	
H5A	1.048177	0.183438	0.733310	0.015*	
H5B	1.178973	0.295883	0.606644	0.015*	
C6	0.82994 (17)	0.38253 (12)	0.62270 (11)	0.0122 (2)	
N1	0.43897 (17)	0.62290 (12)	0.87167 (10)	0.0177 (2)	
H1NA	0.268 (3)	0.582 (2)	0.8186 (19)	0.033 (4)*	
H1NB	0.538 (3)	0.608 (2)	0.800 (2)	0.036 (4)*	
H1NC	0.470 (5)	0.534 (3)	0.953 (3)	0.013 (6)*	0.5
C1	0.47080 (18)	0.81542 (13)	0.93517 (11)	0.0147 (2)	
C2	0.65701 (19)	0.95024 (15)	0.89941 (12)	0.0180 (2)	
H2	0.764255	0.916272	0.830590	0.022*	
C3	0.68658 (19)	1.13477 (14)	0.96421 (12)	0.0179 (2)	
H3	0.814280	1.227114	0.939883	0.021*	

### Atomic displacement parameters (Å^2^)

**Table:**

	*U*^11^	*U*^22^	*U*^33^	*U*^12^	*U*^13^	*U*^23^
O1	0.0309 (4)	0.0199 (4)	0.0270 (4)	0.0138 (3)	−0.0099 (3)	−0.0120 (3)
O2	0.0149 (4)	0.0149 (3)	0.0169 (4)	0.0069 (3)	0.0000 (3)	−0.0023 (3)
C4	0.0124 (4)	0.0124 (4)	0.0173 (5)	0.0051 (3)	0.0023 (3)	−0.0011 (3)
C5	0.0108 (4)	0.0119 (4)	0.0154 (4)	0.0034 (3)	0.0027 (3)	0.0006 (3)
C6	0.0131 (4)	0.0101 (4)	0.0144 (4)	0.0023 (3)	0.0049 (3)	0.0015 (3)
N1	0.0164 (4)	0.0225 (4)	0.0144 (4)	0.0030 (3)	0.0040 (3)	0.0012 (3)
C1	0.0137 (4)	0.0187 (5)	0.0107 (4)	0.0033 (3)	0.0009 (3)	0.0006 (3)
C2	0.0160 (4)	0.0246 (5)	0.0154 (4)	0.0041 (4)	0.0070 (3)	0.0008 (4)
C3	0.0149 (4)	0.0223 (5)	0.0172 (5)	0.0003 (4)	0.0064 (4)	0.0023 (4)

### Geometric parameters (Å, º)

**Table:**

O1—C6	1.2379 (12)	N1—C1	1.4361 (13)
O2—C6	1.2802 (11)	N1—H1NA	0.914 (17)
O2—H2O	1.2197 (6)	N1—H1NB	0.906 (17)
C4—C5	1.5195 (12)	N1—H1NC	0.95 (2)
C4—C4^i^	1.5213 (16)	C1—C2	1.3870 (14)
C4—H4A	0.9900	C1—C3^ii^	1.3916 (13)
C4—H4B	0.9900	C2—C3	1.3876 (14)
C5—C6	1.5123 (12)	C2—H2	0.9500
C5—H5A	0.9900	C3—H3	0.9500
C5—H5B	0.9900		
			
C6—O2—H2O	113.50 (6)	C1—N1—H1NA	111.1 (9)
C5—C4—C4^i^	111.39 (9)	C1—N1—H1NB	111.9 (10)
C5—C4—H4A	109.3	H1NA—N1—H1NB	107.8 (14)
C4^i^—C4—H4A	109.3	C1—N1—H1NC	114.7 (15)
C5—C4—H4B	109.3	H1NA—N1—H1NC	101.0 (18)
C4^i^—C4—H4B	109.3	H1NB—N1—H1NC	109.6 (18)
H4A—C4—H4B	108.0	C2—C1—C3^ii^	120.02 (9)
C6—C5—C4	115.04 (8)	C2—C1—N1	121.06 (8)
C6—C5—H5A	108.5	C3^ii^—C1—N1	118.91 (9)
C4—C5—H5A	108.5	C1—C2—C3	119.97 (9)
C6—C5—H5B	108.5	C1—C2—H2	120.0
C4—C5—H5B	108.5	C3—C2—H2	120.0
H5A—C5—H5B	107.5	C2—C3—C1^ii^	120.01 (9)
O1—C6—O2	123.47 (8)	C2—C3—H3	120.0
O1—C6—C5	120.06 (8)	C1^ii^—C3—H3	120.0
O2—C6—C5	116.47 (8)		
			
C4^i^—C4—C5—C6	−179.82 (9)	C3^ii^—C1—C2—C3	0.09 (16)
C4—C5—C6—O1	172.41 (9)	N1—C1—C2—C3	−179.06 (9)
C4—C5—C6—O2	−8.04 (12)	C1—C2—C3—C1^ii^	−0.10 (16)

Symmetry codes: (i) −*x*+2, −*y*, −*z*+1; (ii) −*x*+1, −*y*+2, −*z*+2.

### Hydrogen-bond geometry (Å, º)

**Table:**

*D*—H···*A*	*D*—H	H···*A*	*D*···*A*	*D*—H···*A*
O2—H2*O*···O2^iii^	1.22	1.22	2.439 (1)	180
O2—H2*O*···O1^iii^	1.22	2.45	3.178 (1)	116
N1—H1*NA*···O1^iv^	0.91 (2)	1.97 (2)	2.871 (1)	171 (1)
N1—H1*NB*···O1	0.90 (2)	2.24 (2)	3.060 (1)	152 (1)
N1—H1*NB*···O2^iii^	0.90 (2)	2.51 (2)	3.098 (1)	123 (1)
N1—H1*NC*···N1^v^	0.95 (2)	1.89 (2)	2.840 (1)	174 (2)

Symmetry codes: (iii) −*x*+1, −*y*+1, −*z*+1; (iv) *x*−1, *y*, *z*; (v) −*x*+1, −*y*+1, −*z*+2.
